# Numerical Investigation of Material Flow and Defect Formation in FRAM-6061 Al Alloy Ring Component Using CEL Simulation

**DOI:** 10.3390/ma19020236

**Published:** 2026-01-07

**Authors:** Yan Ji, Bin Yang

**Affiliations:** Collaborative Innovation Center of Steel Technology, University of Science and Technology Beijing, Beijing 100083, China

**Keywords:** friction rolling additive manufacturing, ring-shape component, numerical simulation, microstructural evolution

## Abstract

In this study, a novel and efficient solid-state additive manufacturing technique, friction rolling additive manufacturing (FRAM), was employed to fabricate an aluminum alloy ring component, significantly reducing process complexity and mitigating solidification defects typical of melt-based techniques. However, previous studies on FRAM have primarily focused on the microstructural characteristics and mechanical properties of flat components, with limited attention paid to ring-shaped components. Owing to the unique geometric constraints imposed during the forming process, ring components exhibit markedly different microstructural evolution and defect formation mechanisms compared with flat counterparts, and these mechanisms remain insufficiently and systematically understood. To address this knowledge gap, the coupled Eulerian–Lagrangian (CEL) method was introduced for the first time to numerically simulate the temperature distribution and residual stress evolution during the FRAM process of ring-shaped components. In addition, tracer particles were incorporated into the simulations to analyze the material flow behavior, thereby systematically elucidating the forming behavior and microstructural evolution characteristics under geometric constraint conditions. Moreover, scanning electron microscopy (SEM) and electron backscatter diffraction (EBSD) were employed to systematically characterize the microstructural evolution and defect morphology. The CEL numerical simulations exhibited good consistency with the experimental observations, demonstrating the reliability and accuracy of the simulation method. The results showed that the peak temperatures were primarily concentrated at the advancing side of the rotation tool, and the temperature on the outer diameter side of the ring was consistently higher than that on the inner diameter side. The lack of shoulder friction on the inner side led to an increased heat dissipation rate, thereby resulting in higher residual stress compared to other regions. The particle analysis revealed that, due to ring geometry, material flow varied across radial regions, resulting in distinct microstructures. Further EBSD analysis revealed that, after the rotating tool passed, the material first developed a preferential orientation with {111} planes parallel to the shear direction, and with more layers, dynamic recrystallization produced an equiaxed grain structure. This study provides a theoretical basis and process reference for the application of the FRAM technique in the manufacturing of large ring components.

## 1. Introduction

In the critical equipment of aerospace, aviation, and marine engineering, various types of annular components are widely employed, such as connecting rings of propellant storage tanks, pipeline systems, and hydraulic accumulators [1,2,3,4,5]. Aluminum alloys, owing to their advantageous material characteristics including low density, high specific strength, and excellent corrosion performance, have become the common material of choice for such annular components. At present, the manufacturing of annular parts mainly relies on traditional forming processes such as ring rolling and integral die forging [5,6,7]. However, with the increasing demand from high-end equipment for components with more complex structures, higher dimensional accuracy, and lighter weight, traditional processes still face considerable challenges in terms of material utilization, dimensional precision, and defect control [8,9]. In recent years, the rapid development of additive manufacturing technologies has provided a new technical pathway for the efficient fabrication of complex annular structures. At present, additive processes used for ring/arc shape components are predominantly melt-based additive manufacturing techniques. such as laser–cold metal transfer (Laser-CMT) hybrid additive manufacturing [10], laser-based additive manufacturing [11], and arc-based additive manufacturing processes [12]. Al alloys are characterized by a high laser reflectivity and a large coefficient of thermal expansion. During melt-based additive manufacturing, these material characteristics make aluminum alloys more susceptible to the formation of metallurgical defects such as residual stresses, component distortion, porosity, and cracking, which in turn lead to the degradation of corrosion performance and mechanical properties and may ultimately result in premature failure [13,14,15]. Consequently, solid-state additive manufacturing technologies for aluminum alloys have attracted increasing attention and have been actively developed in recent years [16,17,18]. Among them, the extensively studied friction stir additive manufacturing (FSAM) process typically produces equiaxed grains only within the stir zone, which can readily lead to an inhomogeneous microstructural distribution across the component cross-section. In contrast, friction rolling additive manufacturing (FRAM) is capable of forming a fully equiaxed microstructure during the deposition process, while also offering advantages such as flexible feedstock configurations and the ability to achieve continuous feeding.

In recent years, the emerging friction rolling additive manufacturing (FRAM) process has achieved notable progress in the fabrication of aluminum alloy ring shape components, with the produced component exhibiting excellent combination of strength and toughness, thereby demonstrating the broad application prospects of FRAM in the forming of large annular structures [19,20]. At present, existing research on FRAM has primarily focused on the flat-plate components. For example, Xie et al. [21] successfully fabricated dense 6061 aluminum alloy flat components using wire feedstock. In addition, they systematically investigated the effect of feedstock form on the microstructure and mechanical properties of FRAM-fabricated components [22]. In terms of numerical simulation, Xie et al. [23] employed the coupled Eulerian–Lagrangian (CEL) method to investigate the evolution of the temperature field and material flow behavior during the FRAM process of flat components. The results indicated that material flow in both the transverse and longitudinal directions promoted effective interlayer bonding during the FRAM process. Liu et al. [24] further explored the effect of tool plunge depth on forming defects and found that with increasing plunge depth, unbonded defects were gradually eliminated.

It is worth noting that the capability of the FRAM process in the fabrication of ring shape components has already been preliminarily validated. However, owing to the unique geometric characteristics of ring-shaped structures, the material flow behavior and temperature field distribution during the additive process inevitably differ significantly from those in conventional flat-plate fabrication, which in turn leads to distinct mechanisms of microstructural evolution that require further in-depth investigation. At the same time, the formation mechanisms of microstructures in the FRAM process, particularly regarding grain orientation and texture evolution still lack systematic research. In addition, the mechanisms underlying defect formation during the FRAM process also remain to be further explored.

Based on the above analysis, this study for the first time employs a coupled Eulerian–Lagrangian (CEL) numerical simulation approach to systematically investigate the temperature distribution characteristics during the formation of ring-shaped components fabricated by friction-based additive manufacturing (FRAM). By introducing tracer particles into the simulation framework, the material flow behavior in three distinct radial regions is revealed, thereby further elucidating the mechanisms of defect formation. In addition, experimental techniques including electron backscatter diffraction (EBSD) and scanning electron microscopy (SEM) are employed to conduct an in-depth analysis of the microstructural characteristics and defect formation mechanisms within the annular components. Through these investigations, this study aims to provide a theoretical basis for the application of the FRAM process in the fabrication of large ring-shaped components, and to offer a scientific foundation for optimizing process parameters and enhancing component performance.

## 2. Experimental Procedures

### Materials and Methods

**Figure 1:**
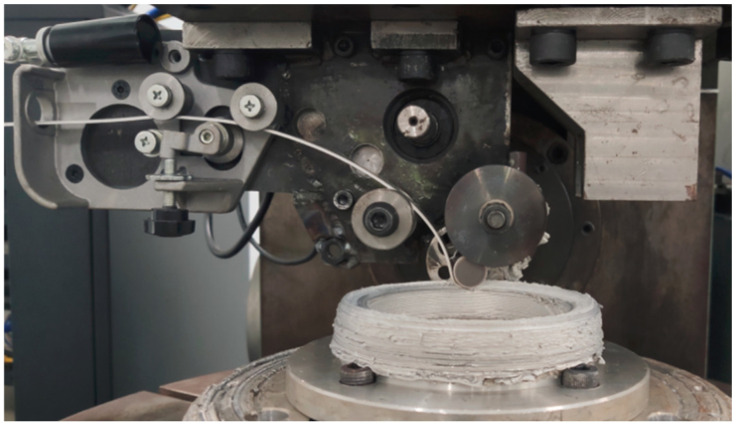
Experimental procedure for the fabrication of ring shape components using FRAM.

The 6061-T0 aluminum alloy strip, with a thickness of 2 mm and a width of 12.5 mm, was used as the feedstock material for the FRAM process in this study, the rotating tool was made of tungsten steel. The chemical composition of the alloy is provided in Table 1, the feedstock was supplied using a self-balancing feeding mechanism. An aluminum alloy ring shape component was fabricated via FRAM process. The component exhibited an inner diameter of 80 mm, an outer diameter of 105 mm, a wall thickness of 12.5 mm, and a height of 48 mm. The experimental procedure for fabricating the ring shape component through FRAM was illustrated in Figure 1.

**Figure 2:**
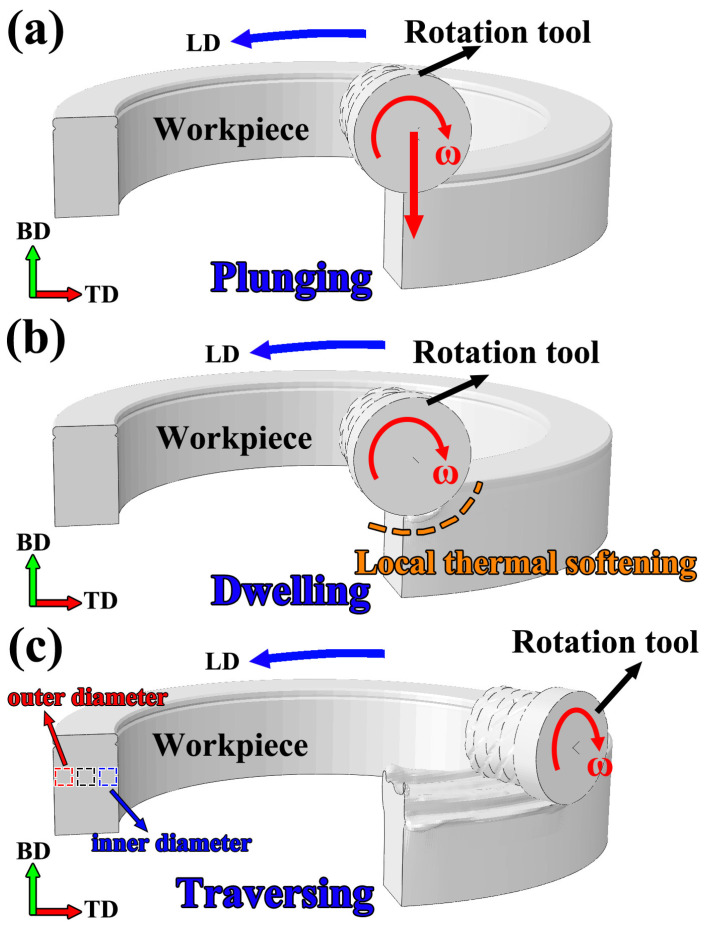
Schematic illustration of the FRAM process: (**a**) plunging stage; (**b**) dwelling stage; (**c**) traversing stage.

As shown in Figure 2, the entire forming process was divided into three stages: the plunging stage, the dwelling stage, and the traversing stage. During the plunging stage, the tool applied a downward displacement at a speed of 1 mm/s in a direction perpendicular to its axis, and the process lasted 2.4 s to reach a plunging depth of 2.4 mm. This was followed by the dwelling stage, during which the tool remained at a constant location for 1 s while maintaining rotation. Finally, in the traversing stage, the tool moved forward along the additive path at a constant speed of 100 mm/min. Throughout the entire process, the rotational speed of the tool was maintained at 900 r/min. 

The specimens for EBSD and SEM analyses were sectioned along planes parallel to the build direction, with the observation surfaces oriented along the BD–TD plane. The specific sampling locations were shown in Figure 2. After sequential grinding with sandpaper, the specimens were subjected to fine polishing with diamond polishing paste, followed by electrolytic polishing using a perchloric acid–ethanol solution. After grinding and polishing, the specimens were electrolytically etched in a solution of HClO_4_-C_2_H_5_OH at room temperature under a voltage of 15 V, in order to reveal the microstructural features. EBSD analysis was performed using a Gemini 450 scanning electron microscope (Carl Zeiss Microscopy GmbH, Oberkochen, Germany) with a step size of 2 μm. SEM morphological observations were conducted with a Supra 55 scanning electron microscope (Carl Zeiss Microscopy GmbH, Oberkochen, Germany). The residual stresses of the FRAM-6061 component were measured using a portable X-ray residual stress analyzer (μ-X360) (Pulstec Industrial Co., Ltd., Hamamatsu, Japan), with measurement locations selected at the inner-diameter side, central region, and outer-diameter side of the annular component. A Cr target was employed as the X-ray source during the measurements, with the tube voltage and tube current set to 30 kV and 1.5 mA, respectively. X-ray residual stress measurement is based on the Bragg diffraction principle of crystalline materials. The fundamental concept is that externally applied loads or internal residual stresses induce slight changes in the interplanar spacing (d-spacing), which in turn lead to corresponding shifts in the diffraction angle (2θ). By applying the sin^2^ψ method to measure and fit the diffraction peak positions at different tilt angles, the lattice strain can be quantitatively converted into the principal stress in a specified direction.

## 3. Establishment of Thermomechanical Coupled Model

### 3.1. Geometric Model and Mesh

**Figure 3:**
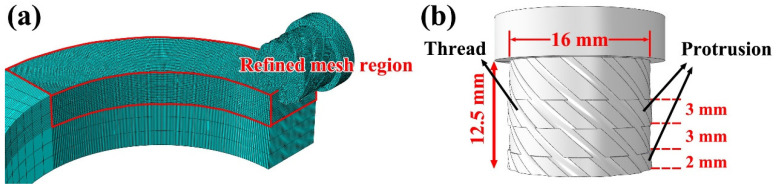
Finite element mesh discretization and geometric characteristics of the rotating tool (**a**) Mesh model of the rotating tool and the workpiece (**b**) Structural dimensions of the rotating tool.

In this study, the rotating tool was modeled using a Lagrangian formulation, while the workpiece was represented within an Eulerian domain to accurately capture severe deformation and material flow. Figure 3 illustrates the meshing strategy of the overall finite element model and the geometric features of the rotating tool. As shown in Figure 3a, local mesh refinement was implemented on the rotating tool and the corresponding contact region of the workpiece to improve the accuracy of the simulation. Figure 3b illustrates the structural model and dimensional parameters of the rotating tool. The tool had a total length of 12.5 mm and featured two protrusions and threaded elements designed to improve material traction, thereby promoting effective material filling and continuous flow during the additive process.

**Figure 4:**
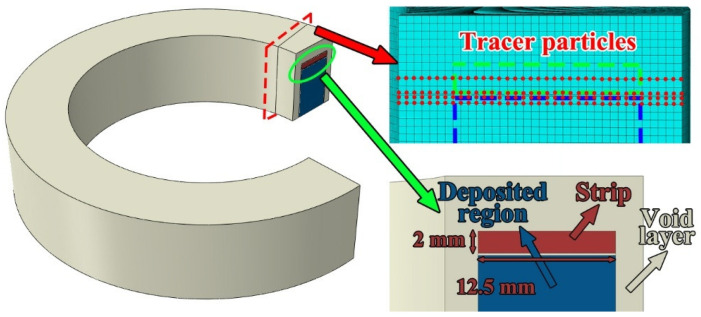
Distribution of tracer particles and void layer.

Figure 4 shows the distribution of tracer particles and the void layer arrangement within the additively manufactured component. To investigate the material flow behavior during the stable advancing stage of the rotating tool, tracer particles were placed at a specified distance ahead of the tool’s initial position. As highlighted by the red box in Figure 4, these particles were distributed at the bottom and central regions of the feedstock strip, as well as on the top region of the previously deposited layer. In the CEL simulation, the feedstock strip was defined with a thickness of 2 mm and a width of 12.5 mm. A void layer was implemented around the outer periphery of the workpiece. Furthermore, a predefined gap of 0.2 mm was introduced between the feedstock strip and the previously deposited region to more accurately capture the relative motion and material filling behavior during the FRAM process.

### 3.2. Material Model and Boundary Conditions

**Figure 5:**
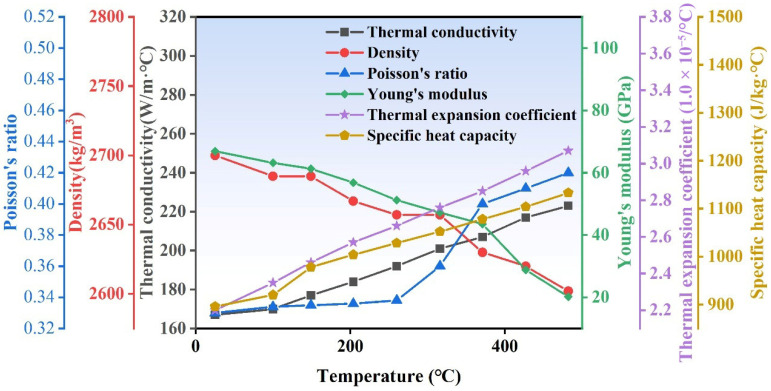
The temperature-dependent material properties of 6061 Al alloy [26].

The temperature-dependent material properties of 6061 aluminum alloy are presented in Figure 5, including density, thermal conductivity, Young’s modulus, coefficient of thermal expansion, and specific heat capacity. Additionally, according to Ref. [23], the friction coefficient between the rotating tool and the material was set to 0.2 in this study.

Before the initiation of FRAM process, both the workpiece and the rotating tool were set at 25 °C. To reasonably describe the heat exchange behavior, the convective heat transfer coefficient between the feedstock strip and the substrate was set to 3000 W/(m^2^·°C), while the coefficient for convection between the workpiece and the surrounding air was set to 300 W/(m^2^·°C). In order to prevent non-physical material loss during the simulation, velocity boundary conditions were applied to all surfaces of the workpiece except the top surface, constraining their velocity components in the X, Y, and Z directions (V_X_ = V_Y_ = V_Z_ = 0).

In this study, the Johnson-Cook (J-C) constitutive model was adopted to characterize the work hardening effect, strain rate hardening effect, and high-temperature softening effect of the plate material during the FRAM process. The J-C constitutive equation can be used to describe the rheological behavior of materials under conditions of large plastic deformation, high strain rates, and elevated temperatures. Its flow stress expression is as follows:

(1) $$\sigma =\left(A+B\varepsilon_{p}^{n}\right)\left[1+C\ln\left(\frac{{\dot{\varepsilon }}_{p}}{{\dot{\varepsilon }}_{0}}\right)\right]\left[1-{\left(\frac{T-T_{r}}{T_{M}-T_{r}}\right)}^{m}\right]$$

Here A, B, n, C, and m are material constants; εp denotes the equivalent plastic strain; ${\dot{\varepsilon }}_{p}$ represents the equivalent plastic strain rate; ${\dot{\varepsilon }}_{0}$ is the reference strain rate of 1 s^−1^; Tr is the room temperature; and Tm is the melting point of the aluminum alloy. The parameters of the Johnson-Cook model adopted in this study are listed in Table 2.

To reduce the computational complexity and focus on the primary thermo-mechanical behavior of the FRAM process, several simplifications were adopted [28]:

(1) In this study, the contact heat transfer between the workpiece and the backing plate was neglected, and the contact heat transfer between the workpiece and the platen was simplified as convective heat transfer.
(2) The friction coefficient was simplified as a constant value.
(3) The sheet and substrate materials were considered homogeneous and isotropic, while the tool tip was assumed to be a rigid body with incompressible volume.

## 4. Result and Discussion

### 4.1. Numerical Simulation

#### 4.1.1. Temperature Distribution Characteristics

**Figure 6:**
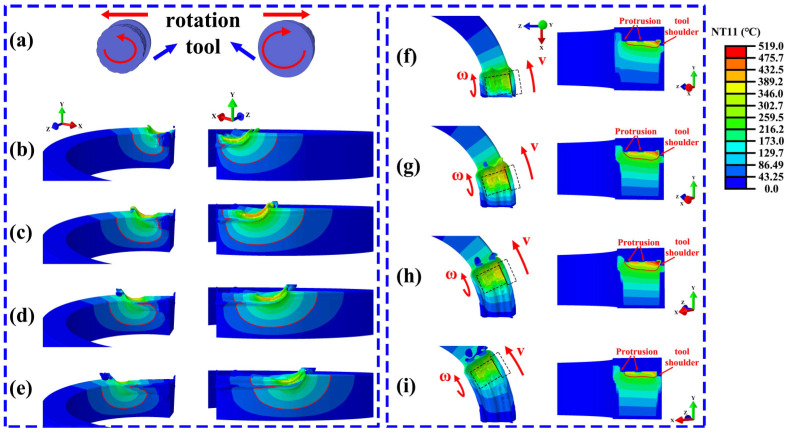
Temperature distribution characteristics during the FRAM process (**a**–**e**) Temperature profiles on the inner- and outer-diameter sides (**f**–**i**) Temperature distributions on the top surface and the TD-BD cross-section.

Figure 6 illustrates the evolution of the temperature distribution at different time points during the steady advancing stage of the rotating stirring tool. Specifically, Figure 6a shows the relative movement of the stirring tool with respect to the annular additively manufactured component. Figure 6b–e correspond to the instantaneous temperature field distributions inside and outside the component at 4 s, 8 s, 12 s, and 16 s of the additive process, respectively. Figure 6f–i presented the surface and cross-sectional temperature distributions of the component at these corresponding time points.

From Figure 6b–e, it can be observed that during the advancing process of the stirring tool, the temperature contour behind the tool exhibits a distinct concave feature, indicating that the heat input in this region is relatively low, while the highest temperatures are consistently located in the region ahead of the tool. This phenomenon suggests that during continuous material advancement and plastic deformation, heat accumulation is primarily concentrated in the area in front of the stirring head.

As shown in Figure 6f–i, due to the frictional heat generated between the tool shoulder and the base material, the higher temperatures are mainly distributed on the shoulder side of the stirring tool. This temperature distribution characteristic is similar to that observed in FSW and FSW-based solid-state additive manufacturing processes. Throughout the entire additive process, the temperature on the outer side of the component consistently remains higher than that on the inner side.

**Figure 7:**
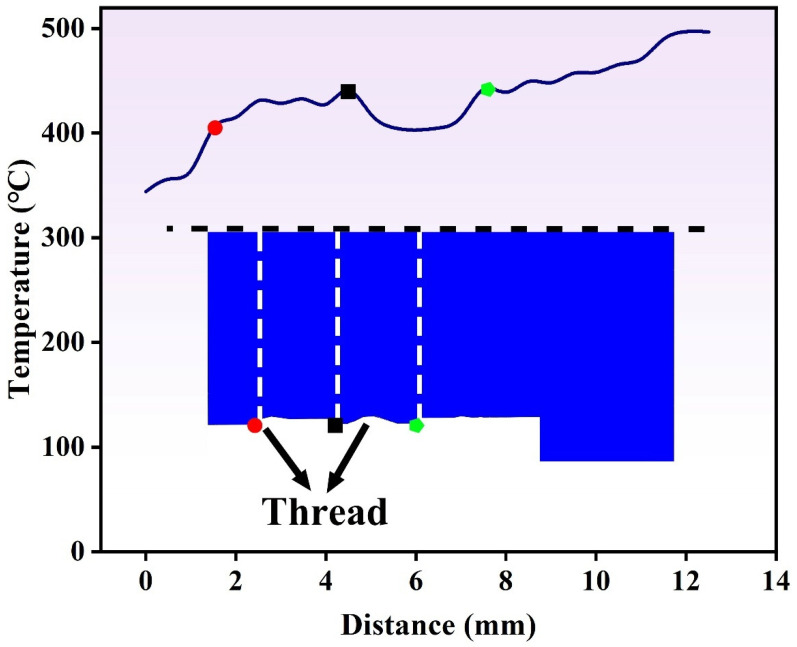
Temperature distribution at 6.3 s during the traversing stage of the rotating tool.

Figure 7 shows the temperature distribution when the rotating tool had advanced steadily to 6.3 s, along with the cross-sectional state of the tool at that moment. The horizontal axis denotes the distance from the bottom of the tool, while the vertical axis represents the corresponding local temperature. Overall, a gradual increase in temperature was observed along the radial direction from the inner toward the outer side. At both ends of the protrusions on the rotating tool, localized temperature rises are observed, forming distinct heat accumulation zones. Notable localized temperature elevations appeared at both ends of the protrusions on the rotating tool, indicating pronounced thermal accumulation zones. Additionally, the temperature distribution in these regions exhibited a degree of fluctuation, which is likely associated with the presence of the threaded geometry. The elevated temperatures observed at both the protrusion ends and threaded sections were primarily attributed to the complex geometry, which promoted localized shear intensification and concentrated plastic deformation, thereby enhancing heat generation and resulting in thermal accumulation in these areas.

#### 4.1.2. Stress Distribution Characteristics

**Figure 8:**
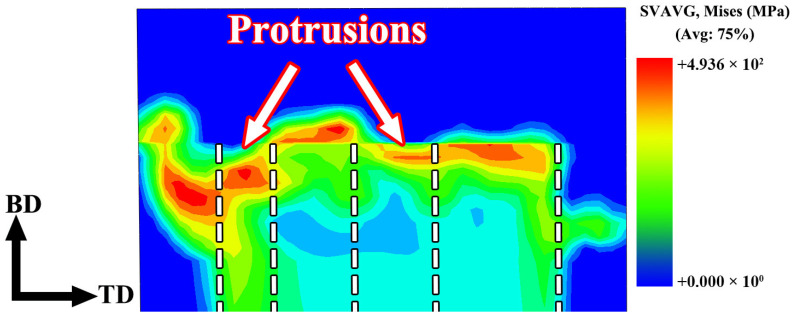
Equivalent residual stress distribution map of FRAM-6061.

Due to the difference in the operating mode of the rotating tool, the FRAM process exhibits distinct characteristics compared with other FSW-based solid-state additive manufacturing techniques. This difference not only led to a significantly different temperature field distribution but also altered the evolution behavior of the stress field during the FRAM process. Figure 8 shows the equivalent residual stress distribution of the FRAM-6061 component on the BD-TD plane. As shown in Figure 8, a certain degree of stress concentration was observed beneath the region affected by the rotating tool, with a more pronounced concentration occurring on the inner-diameter side (near the bottom of the rotating tool). Although the temperature field analysis indicated that the temperature in this region was lower than that at the central and outer-diameter sides, the lack of mechanical constraint and thermal input from the tool shoulder at the bottom resulted in a faster local cooling rate and a larger thermal gradient, thereby producing a higher residual stress distribution in this area. It should be noted that the residual stress was not solely induced by temperature gradients and cooling rates; plastic deformation also constituted a significant contributor to its formation. The CEL numerical simulation employed in this study represented a thermomechanical coupled analysis, and the resulting stress field inherently reflected the combined effects of thermal loading and plastic deformation.

#### 4.1.3. Material Flow Simulation

**Figure 9:**
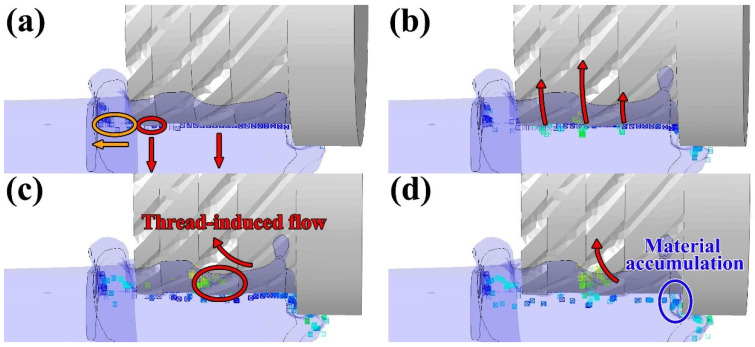
Tracer particles distributions at different times (**a**) 4 s (**b**) 4.8 s (**c**) 5.6 s (**d**) 6.4 s.

Due to the distinctive operational characteristics of the rotating tool in the FRAM process, the material flow behavior exhibits significant differences compared to those observed in other solid-state additive manufacturing methods. To further elucidate the formation mechanisms of the microstructures in different regions, tracer particles were introduced into the CEL simulation to track the material flow behavior throughout the FRAM process. Figure 9 presents the motion trajectories of the tracer particles at various time steps (4 s, 4.8 s, 5.6 s, 6.4 s) during the steady advancing stage of the stirring tool.

It can be seen from Figure 9 that, as the stirring tool advances, the tracer particles located beneath the protrusion region are the first to undergo downward movement. Subsequently, the particles in the non-protrusion regions also gradually migrate downward and continue to flow toward the rear of the rotating tool. Notably, the material located near the edge regions on both sides of the protrusions exhibited a preferential rearward flow behavior. As the rotating tool continued to advance, the material exhibited a tendency to flow inward due to the influence of the threaded structure. This inward flow became more pronounced at the bottom of the tool, where the absence of the shoulder structure led to reduced confinement of the material. In contrast, at the outer edge of the ring-shaped component, the combined constraint imposed by the tool shoulder and the curved geometry of the workpiece restricted material flow, resulting in localized accumulation near the outer edge.

**Figure 10:**
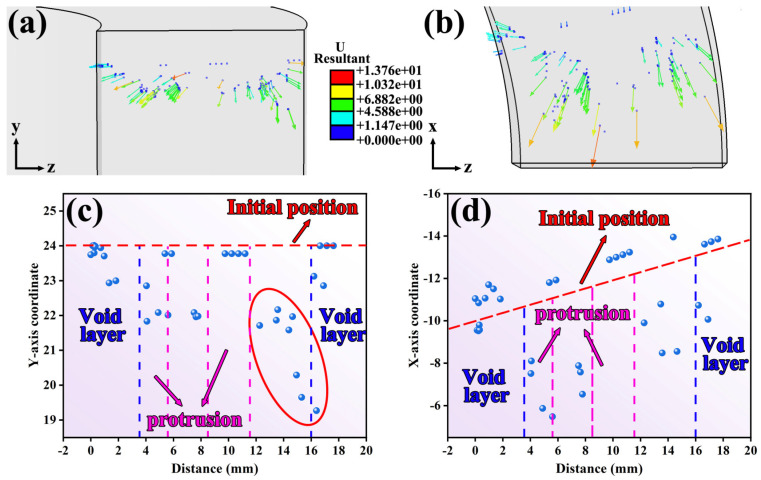
Tracer particle distributions and corresponding coordinates within the feedstock strip at 6.4 s during the traversing stage of the rotating tool (**a**) Tracer particle distribution on the TD-BD cross-section (**b**) tracer particle distribution on the LD-TD cross-section (**c**) coordinate distribution of tracer particles along the BD direction (**d**) coordinate distribution of tracer particles along the LD direction.

**Figure 11:**
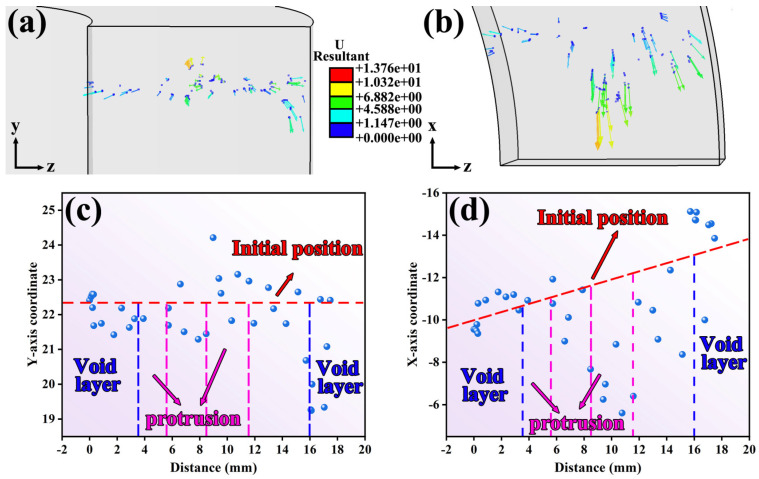
Tracer particle distributions and corresponding coordinates within the deposited layer at 6.4 s during the traversing stage of the rotating tool (**a**) Tracer particle distribution on the TD-BD cross-section (**b**) tracer particle distribution on the LD-TD cross-section (**c**) coordinate distribution of tracer particles along the BD direction (**d**) coordinate distribution of tracer particles along the LD direction.

To further elucidate the flow characteristics of the additive layer and the previously deposited material during the additive process, the positions of tracer particles within both regions were extracted at 6.4 s during the steady-state advancing stage of the rotating tool, as shown in Figure 10 and Figure 11.

Figure 10a,b and Figure 11a,b respectively illustrated the spatial distributions of tracer particles within the additive layer and the previously deposited layer on the TD-BD and TD-LD planes at 6.4 s. Figure 10c,d and Figure 11c,d, further presented the particle coordinate distributions in the BD (*Y*-axis) and LD (*X*-axis) directions, where the horizontal axis in each plot denoted the distance from the bottom of the rotating tool. It should be noted that, due to the ring-shaped geometry of the workpiece, the tracer particles were arranged radially, resulting in a distribution path that formed an angle with the *X*-axis. Consequently, in Figure 10d and Figure 11d, their initial coordinates in the LD direction gradually increased with increasing horizontal coordinate.

As shown in Figure 10, the overall material flow was predominantly directed toward the rear side of the rotating tool. In the inner-diameter region, owing to the relatively weak geometric constraint imposed by the tool shoulder and the annular geometry of the component, the translational velocity of the rotating tool was comparatively low. As a result, the tracer particles in this region exhibited a pronounced tendency to accumulate toward the inner diameter, with inward migration being significantly more pronounced than migration toward the rear side of the tool. By contrast, tracer particles in the central region displayed a stronger tendency to migrate toward the rear side of the rotating tool. At the outer-diameter edge, the relatively larger radial space allowed some particles to exhibit a slight outward migration trend, which showed a slight variation from the tracer particle analysis results obtained from CEL simulations of FRAM plate components [24]. Moreover, the presence of the shoulder structure imposed kinematic constraints on material flow in this region, resulting in a noticeable downward inclined motion of the tracer particles. This flow behavior was consistent with the microstructural variations observed at the outer-diameter edge relative to other regions.

In the previously deposited layer shown in Figure 11, the distribution of tracer particles closely resembled that observed in the feedstock strip in Figure 10. Moreover, as shown in Figure 11c, the material in the lower layer exhibited an upward movement tendency along the BD direction at 6.4 s, this behavior indicates that effective interlayer mixing occurred between the upper and lower layers during the FRAM process, which contributed to enhancing interfacial bonding quality and consequently reduced the risk of typical solid-state additive manufacturing defects such as unbonded defects.

**Figure 12:**
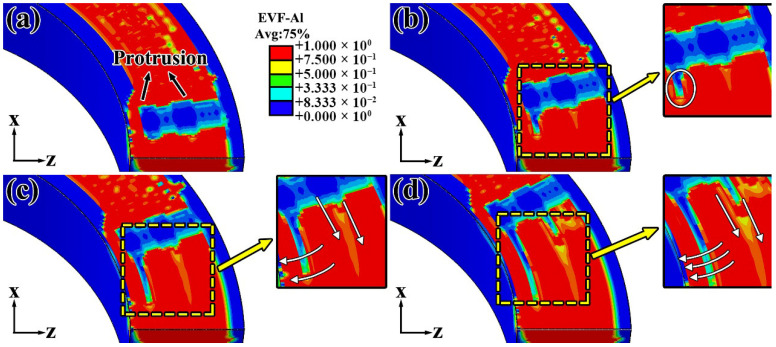
Material flow evolution at different time (**a**) 4.8 s (**b**) 8 s (**c**) 12 s (**d**) 16 s.

Figure 12 presents the Eulerian Volume Fraction (EVF) distributions at the interface between the feedstock strip and the previously deposited layer at different time points (4.8 s, 8 s, 12 s, and 16 s), illustrating the evolution of material distribution during the FRAM process. Here, EVF = 1 corresponds to regions fully occupied by aluminum alloy, whereas EVF = 0 represents void layers that remained unfilled. Figure 12 facilitates a more intuitive analysis of the material filling evolution during the FRAM process.

Under the rotating tool, the material experienced a significant temperature rise due to the combined effects of frictional heating and severe plastic deformation, thereby promoting plastic flow. Owing to the presence of protrusions of the tool, the region directly beneath these features initially exhibited a low EVF, appearing as blue zones in the simulation. As the tool continued to rotate and advance, void regions first appeared behind the protrusion at the bottom of the tool, primarily due to material loss toward the inner-diameter side. Meanwhile, a slight decrease in EVF was also observed behind the central protrusion, indicating that material in this area predominantly flowed rearward along the tool path, allowing for timely and effective filling. As the deposition process progressed, material near both ends of the tool’s bottom protrusion tended to migrate toward the inner diameter, gradually filling the previously unfilled voids. These variations in local filling mechanisms resulted in distinct morphologies of interfacial unbonded defects across various regions.

**Figure 13:**
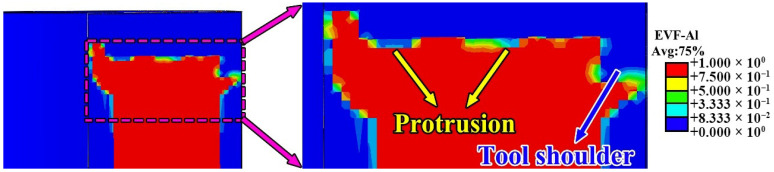
Simulation results of void defects on the TD-BD cross-section.

Figure 13 illustrates the EVF distribution on the BD-LD cross-section, revealing the spatial characteristics of void defects in a plane perpendicular to the TD, in the EVF map, blue regions correspond to unfilled void layers, whereas red regions denote areas that have been fully occupied by aluminum alloy. As shown in Figure 13, void defects tend to form beneath the protrusions of the rotating tool, consistent with the trends observed in Figure 12. Based on the results in Figure 12, it can be inferred that the interfacial unbonded defects near the inner-diameter side primarily result from excessive material flow toward the inner periphery, which causes insufficient backfilling in the region behind the rotating tool. In contrast, the material located beneath the central protrusion of the tool initially experienced a downward compressive force along the BD. As the tool continues to advance, the plastically deformed material flows rearward and accumulated, resulting in more sufficient filling in this region and thus a lower propensity for void formation. This behavior is consistent with the material flow and filling mechanisms revealed in Figure 9, Figure 10, Figure 11 and Figure 12. Regarding the outer-diameter side, tracer particle analysis revealed that, due to the geometric constraints imposed by the component, the material in this region exhibited a downward-inclined migration pattern. During this process, interfacial unbonded defects could form if the descending flow failed to completely fill the resulting local gaps.

### 4.2. Experimental Verification

#### 4.2.1. Residual Stress

The development of residual stress is related to the temperature gradient and cooling rate. Thus, its distribution reflects the combined effects of the thermo-mechanical coupling process. By comparing the simulated and experimentally measured residual stress distributions, the validity and accuracy of the developed numerical model in predicting the evolution of the temperature and stress fields were indirectly verified.

**Figure 14:**
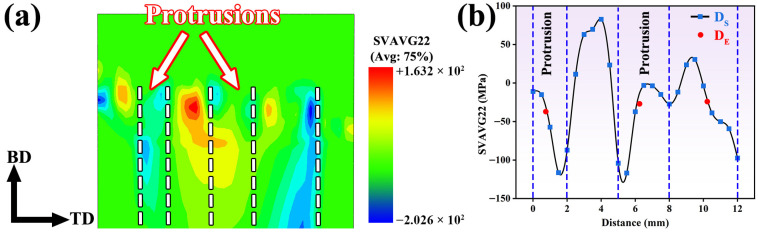
Longitudinal residual stress of the FRAM-6061 component on the BD-TD section (**a**) Distribution of longitudinal residual stress from simulation; (**b**) Comparison between simulation and experimental results.

Considering that the component fabricated in this study through the FRAM process possessed an annular geometry, a certain degree of deviation may exist between the measurement orientations used for the residual stress evaluation and the coordinate system employed in the numerical simulation. However, the BD direction at all measurement locations remained consistent. Therefore, the BD-TD plane was selected as the measurement cross-section, and the residual stresses along the BD direction were measured at the inner-diameter, central, and outer-diameter regions, as shown in Figure 14.

Figure 14a presents the distribution of residual stress on the BD-TD plane of the FRAM-6061 component, while Figure 14b compared the simulated stress data (D_S_) with the experimentally measured data (D_E_) along the radial direction from the inner to the outer diameter. As shown in Figure 14a, compressive residual stress was predominantly observed beneath the protrusions of the rotating tool, while tensile stress was present between the protrusions. This phenomenon can be attributed to the pronounced compressive action exerted by the protruded regions of the rotating tool, which induces compression-dominated deformation along the build direction, thereby resulting in a compressive residual stress state. In contrast, the material located between the two protrusions is subjected to traction from the adjacent protruded regions under geometric constraint, and the deformation compatibility among neighboring regions promotes tensile deformation along the build direction, leading to the formation of tensile residual stress. Additionally, relatively high compressive stress was observed on the outer-diameter side near the shoulder-affected region. The experimentally measured residual stresses at the inner-diameter, central, and outer-diameter regions were −35 MPa, −27 MPa, and −24 MPa, respectively, which indicated close consistency with the corresponding simulation results. This result indicated that the established model was capable of accurately capturing the residual stress distribution characteristics of the component.

#### 4.2.2. Microstructure and Defect Characterization

**Figure 15:**
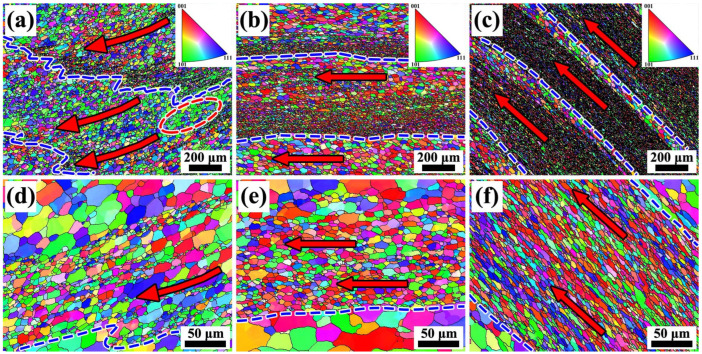
Microstructural characteristics of different regions (**a**–**c**) IPF maps of the inner-diameter edge, central, and outer-diameter edge regions (**d**–**f**) magnified views corresponding to regions (**a**–**c**).

**Figure 16:**
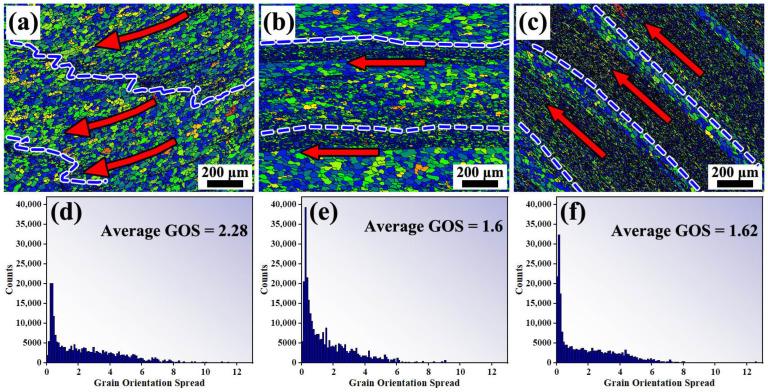
Recrystallization degree in different regions (**a**–**c**) GOS maps of the inner-diameter edge, central region, and outer-diameter edge (**d**–**f**) corresponding GOS distribution histograms for the inner-diameter edge, central region, and outer-diameter edge.

To further systematically investigate the variation in microstructural characteristics across different regions, the inverse pole figure (IPF) maps and grain orientation spread (GOS) maps corresponding to various radial positions are presented in Figure 15 and Figure 16. The results indicate that the differences in microstructural morphology between the central region of the annular component and its inner- and outer-diameter edge regions. These differences resulted from the coupled effects of the material flow paths and temperature variations experienced by each region during the additive manufacturing process. In friction stir-based additive manufacturing processes, temperature elevation generally promotes the occurrence of recrystallization [29]. For high stacking fault energy metals such as aluminum alloys, the dominant recrystallization mechanism is Continuous Dynamic Recrystallization (CDRX) [30]. Figure 15 present the IPF maps of the inner-diameter edge region, the central main region, and the outer-diameter edge region, respectively. From Figure 15, it can be observed that in the central region of the component, due to repeated stirring, the microstructure exhibits a characteristic alternating distribution of coarse grains in the intra-layer regions and fine grains in the inter-layer regions, with relatively straight interfaces between the coarse- and fine-grain bands. At the inner-diameter edge region, although a similar alternating coarse-fine grain feature is present, the number of fine grains is relatively smaller, and the structure exhibits a pronounced flow tendency toward the inner diameter. In contrast, at the outer-diameter edge, unlike the central region, the fine-grain bands are wider, and the grains exhibit a flow trend inclined upward. The formation mechanism of this feature is consistent with the tracer particle analysis results revealed in Section 4.1.3.

The GOS map reflects the degree of grain distortion by calculating the deviation of all points within a grain relative to its average orientation, namely the intragranular orientation distortion, thereby enabling the evaluation of the extent of recrystallization [31]. Generally, a low GOS value (≤1–2°) indicates that the grain has undergone complete recrystallization, whereas a higher GOS value suggests that the grain remains in a deformed or subgrain state. Figure 16 show that different degrees of recrystallization occurred in all regions during the FRAM process, According to the temperature distribution shown in Figure 6, the temperature gradually increases from the inner-diameter side to the outer-diameter side along the radial direction, and under this temperature distribution condition, the central region and the outer-diameter edge region exhibiting more pronounced recrystallization, while the inner-diameter side, due to its relatively lower temperature, displayed a slightly reduced overall degree of recrystallization. In this region, some low-angle grain boundaries (LAGBs) that had not yet transformed into high-angle grain boundaries (HAGBs) can still be observed in Figure 15, further confirming that the microstructural evolution during the friction rolling additive process is predominantly governed by the mechanism of CDRX.

**Figure 17:**
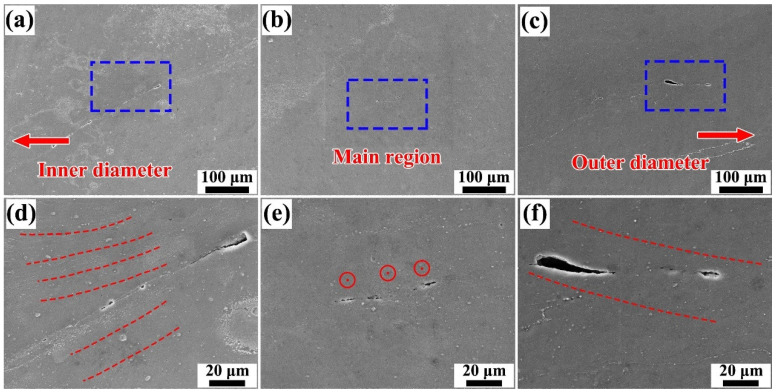
Morphological characteristics of unbonded defects in different regions. (**a**) inner diameter edge region (**b**) central region (**c**) outer diameter edge region (**d**) magnified view of the blue-boxed area in (**a**,**e**) magnified view of the blue-boxed area in (**b**,**f**) magnified view of the blue-boxed area in (**c**).

The SEM images of unbonded defects in the three regions are presented in Figure 17, where Figure 17a–c correspond to void defects at the inner-diameter side, the central region, and the outer-diameter side, respectively, whereas Figure 17d–f present magnified views of the areas highlighted by the blue boxes. As shown in Figure 17a,d, the inner-diameter side primarily exhibits material flow toward the inner diameter with insufficient compensation in the BD-direction, resulting in the formation of linearly distributed unbonded defects along the inner edge. The defects observed in the central region were primarily attributed to insufficient material backfilling after the advancement of the rotating tool. When the material failed to backfill and fully occupy this region, thereby generating unbonded defects. The void defects on the outer-diameter side exhibited a downward-inclined morphology, which was closely associated with the downward-inclined material flow in this region induced by the action of the rotating tool and the geometric constraints of the component. Overall, the unbonded defects on both the outer- and inner-diameter sides were mostly concentrated near the edge regions. Nevertheless, the FRAM component exhibited a high overall density, with only a few small defects detected, all with widths of approximately 1 μm.

### 4.3. Mechanism of Texture Formation of FRAM

To investigate the microstructural evolution of the material under the thermo-mechanical effects of subsequent layers during the multi-layer additive process, a spatial-temporal equivalence approach was employed. In this method, the topmost layer of the cross-section was regarded as the initial state immediately after deposition, while the second layer from the top was considered to represent the state of the first layer after one additional deposition, and so forth. By comparing the grain orientations and grain boundary characteristics at different depths, the evolution pathway of the microstructure was elucidated.

**Figure 18:**
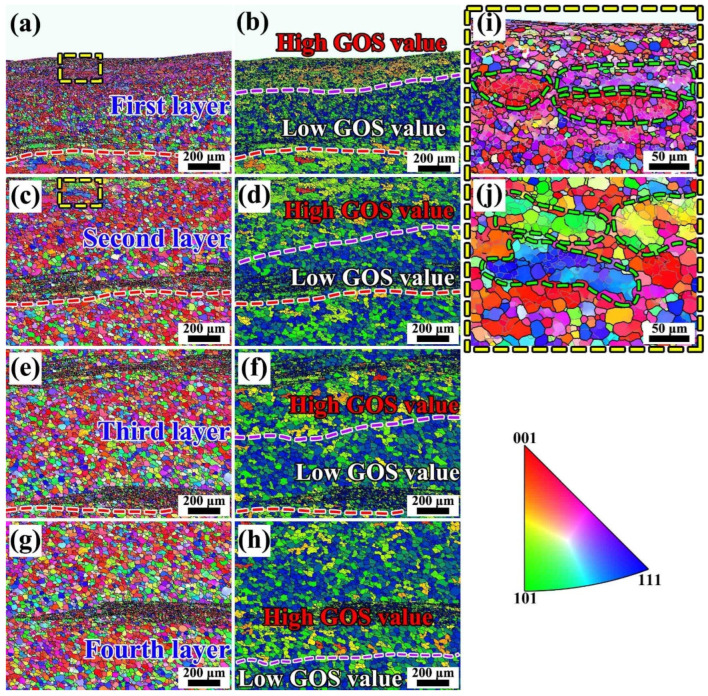
IPF and GOS results of the top four layers of the FRAM component (**a**,**c**,**e**,**g**) IPF maps of the first to fourth layers from top to bottom (**b**,**d**,**f**,**h**) GOS maps of the first to fourth layers from top to bottom (**i**) magnified view of the yellow-boxed area in (**a**,**j**) magnified view of the yellow-boxed area in (**c**).

**Figure 19:**
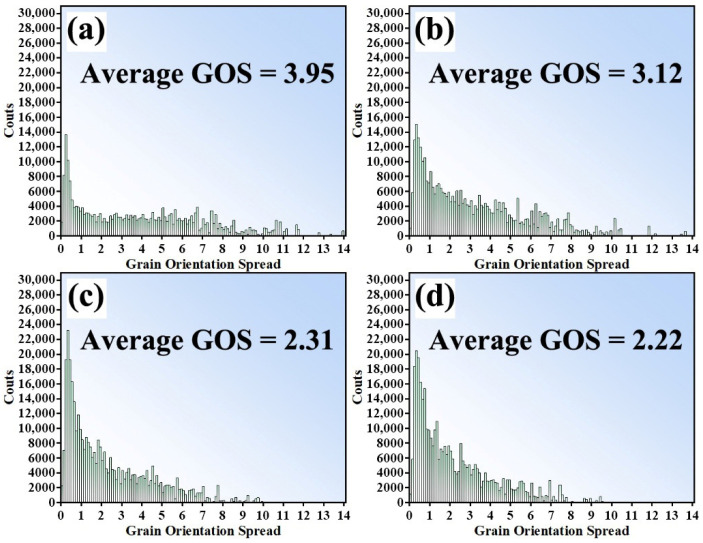
Histograms of GOS distribution for the top four layers (**a**) First layer (**b**) Second layer (**c**) Third layer (**d**) Fourth layer.

The IPF maps and GOS maps corresponding to the four surface-near layers of the top region are shown in Figure 18. Specifically, Figure 18a,c,e,g present the IPF maps of these four layers, while Figure 18b,d,f,h display the corresponding GOS maps. Figure 19 presents the histogram distributions of grain orientation spread (GOS) values for these four regions. From the IPF map in Figure 18a, it can be seen that the top region of the first additive layer, subjected to strong shear stress, experienced severe plastic deformation, with some grains containing numerous low-angle grain boundaries (LAGBs), indicating the presence of abundant subgrain structures. In contrast, the grain orientations in the bottom region of the first additive layer tended to be more randomly distributed, and no obvious presence of large grains containing numerous LAGBS were observed. As the additive process continues (Figure 18c), the accumulated thermal input promoted recrystallization in the grains of the previously additive layer, reflected in the increasingly random grain orientations and the reduction in GOS values, as shown in Figure 19, indicating an increasing degree of recrystallization. Further analysis of Figure 18e,g reveal that the LAGBs within the initially coarse grains gradually transformed into high-angle grain boundaries (HAGBs). Meanwhile, the corresponding regions exhibit a continuous decrease in GOS intensity, reflecting a typical continuous dynamic recrystallization (CDRX) process.

**Figure 20:**
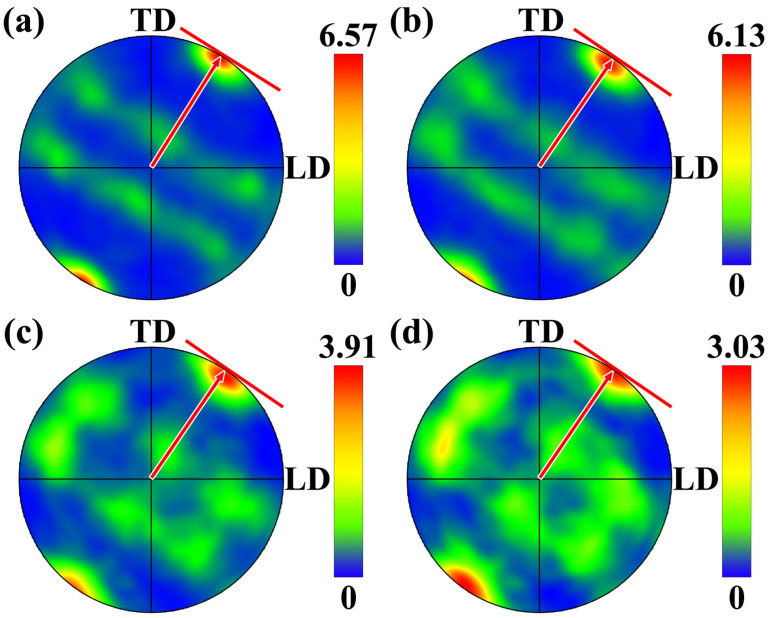
{111} pole figures for the top four layers (**a**) First layer (**b**) Second layer (**c**) Third layer (**d**) Fourth layer.

Figure 20 presents the {111} pole figures corresponding to the four regions shown in Figure 18. From these pole figures, it can be observed that distinct concentrated poles were present, indicating that the grains exhibit a certain degree of preferred orientation. As the number of additive layers increased, the pole distributions gradually become more dispersed, and the corresponding texture intensity weakened, reflecting the occurrence and progression of recrystallization. Further analysis revealed that the {111} poles were mainly distributed within the LD-TD plane, with concentration around a direction approximately 60° from the TD orientation. Literature studies have demonstrated that, in face-centered cubic (FCC) materials, the {111} close-packed planes tend to align parallel to the direction of shear when subjected to intense shear deformation [32,33]. As seen in the tracer particles simulation results in Figure 9, Figure 10 and Figure 11, influenced by the coupling of the component’s geometric morphology with the protrusion and threaded structure of the rotating tool, the material primarily flowed along the rear of the tool and toward the inner diameter during deposition. This caused the shear direction lying within the LD-TD plane and form a certain angle with the TD direction.

**Figure 21:**
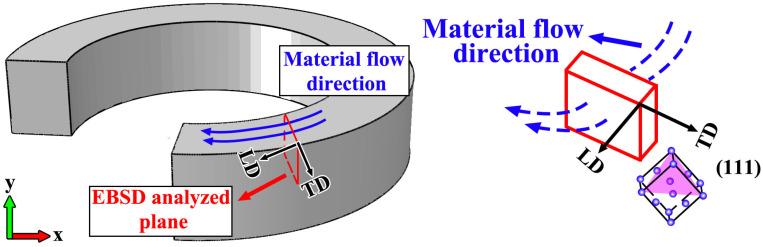
The schematic diagram of crystal orientation of top layer.

A schematic illustration of the spatial relationship between the shear stress direction and the TD direction is provided in Figure 21 to facilitate understanding of the correlation between texture evolution and stress state. The observation plane for the pole figures was sectioned along the radial direction of the ring-shaped component and oriented perpendicular to the BD direction, corresponding to the LD-TD plane. According to the tracer particles analysis results, during the FRAM process, the material did not accumulate entirely behind the rotating tool but instead exhibited a tendency to migrate toward the inner-diameter side, resulting in the shear direction forming a certain angle with the TD direction. Due to the {111} planes tended to align parallel to the shear direction, a spatial orientation relationship as shown in Figure 21 was established, which consequently led to the {111} pole distribution pattern observed in Figure 20.

## 5. Conclusions

In this study, the temperature field distribution and microstructural evolution mechanisms during the FRAM process for fabricating ring component were clarified using CEL numerical simulations combined with EBSD results. The main conclusions are as follows:

1. During the FRAM process for fabricating ring component, the peak temperature was primarily concentrated in the front region of the rotating tool along the advancing direction. Moreover, the more pronounced friction between the tool shoulder and the outer-diameter side of the ring resulted in the temperature on the outer-diameter side remaining consistently higher than that on the inner-diameter side throughout the additive process. The simulation results of residual stress revealed that, due to the absence of frictional effect with the tool shoulder on the inner-diameter side, the cooling rate in this region increased, resulting in higher residual stress compared to other regions. The measured residual stress along the BD indicated close consistency with the corresponding simulation results, thereby indirectly validating the reliability and applicability of the thermo-mechanical coupling model established in this study.
2. Tracer particle analysis revealed that, during the FRAM process of ring components, the material initially flowed downward along the build direction and subsequently accumulated behind the rotating tool. The material tended to migrate toward the inner-diameter side under the combined effect of tool threads and the geometric constraints imposed by the ring. Notably, in the outer-diameter edge region, the constraints imposed by the tool shoulder and the ring geometry produced a pronounced inclined downward flow, leading to microstructural variations between the edge and central regions.
3. EVF analysis further elucidated the material filling mechanism during the FRAM process of ring component. As the material accumulated behind the rotating tool, the material in the inner-diameter and central regions exhibited a tendency to migrate toward the inner-diameter side, with this trend being more pronounced in the inner-diameter region due to the absence of shoulder constraint. In contrast, in the outer-diameter edge region, the combined action of the tool shoulder and the ring geometry caused the material to accumulate behind the tool and to exhibit a pronounced downward flow along an inclined path. These distinct flow patterns ultimately resulted in variations in the morphology of unbonded defects across different regions.
4. Describing the temporal evolution process through the layer-by-layer deposition sequence along the build direction, the microstructural evolution of the first deposited layer under different deposition sequences was systematically investigated. The results demonstrated that, during the deposition of the first layer, intense shear deformation in the top region led to the development of a pronounced texture with {111} planes aligned parallel to the shear direction, accompanied by a high density of subgrain structures within the grains. As additional layers were deposited, the temperature rise promoted continuous dynamic recrystallization in the underlying grains, gradually transforming the microstructure into a fully recrystallized equiaxed grain structure, and with the deposition of the fourth layer, the recrystallization process was complete.

## Figures and Tables

**Table 1:** Chemical composition (wt.%) of AA6061 (GB/T 3190-2020 [25]) used in this study.

Cu	Mn	Mg	Zn	Cr	Ti	Si	Fe	Al
0.15~0.4	0.15	0.8~1.2	0.25	0.04~0.35	0.15	0.4~0.8	0.7	Bal.

**Table 2:** Johnson-Cook plasticity model constants for 6061-Al alloy [27].

A (MPa)	B (MPa)	C	n	m	T_r_ (°C)	T_m_ (°C)
324	114	0.002	0.42	1.34	25	583

## Data Availability

The original contributions presented in this study are included in the article. Further inquiries can be directed to the corresponding author.
